# Arterial Stiffness, Sugar-Sweetened Beverages and Fruits Intake in a Rural Population Sample: Data from the Brisighella Heart Study

**DOI:** 10.3390/nu11112674

**Published:** 2019-11-05

**Authors:** Arrigo F.G. Cicero, Federica Fogacci, Giovambattista Desideri, Elisa Grandi, Elisabetta Rizzoli, Sergio D’Addato, Claudio Borghi

**Affiliations:** 1 Hypertension and Atherosclerosis Research Group, Medical and Surgical Sciences Department; Sant’Orsola-Malpighi University Hospital, Building 2–IV Floor, Via Albertoni 15, 40138 Bologna, Italy; federicafogacci@gmail.com (F.F.); elisa.grandi@unibo.it (E.G.); elisabetta.rizzoli@unibo.it (E.R.); sergio.daddato@unibo.it (S.D.); claudio.borghi@unibo.it (C.B.); 2 Department of Life, Health and Environmental Sciences, University of L’Aquila, Coppito, 67100 L’Aquila, Italy; giovambattista.desideri@univaq.it

**Keywords:** fruits, sugar-sweetened drinks, serum uric acid, arterial stiffness, pulse wave velocity

## Abstract

Introduction: There is conflicting information linking fruit and fructose intake with cardiometabolic disorders. The main objective of our study was to evaluate the association between intake of fruits and sugar-sweetened beverages, and carotid-femoral pulse wave velocity (cfPWV), a non-invasive marker of arterial aging, in a large population sample. Methods: For this study, we selected four age and sex-matched subgroups from the last Brisighella Heart Study population survey, after exclusion of those in secondary prevention for cardiovascular diseases, affected by gout and moderate-to-severe chronic kidney disease (defined as eGFR < 60 mL/min), and/or actively treated with direct vasodilating drugs (calcium-antagonists, alpha-blockers, nitrates). The remaining subjects were classified into four groups: (1) low fruit and low sugar-sweetened beverage intake (LFLB), (2) high fruit and low sugar-sweetened beverage intake (HFLB), (3) low fruit and high sugar-sweetened beverage intake (LFHB), (4) high fruit and high sugar-sweetened beverage intake (HFHB). Results: CfPWV was significantly elevated in subjects consuming a higher fructose load, particularly when it was derived from industrially sweetened beverages (pooled LFHB & HFHB: 9.6 ± 2.3 m/s; pooled LFLB & HFLB: 8.6 ± 2.3 m/s, *p* < 0.001). Moreover, the main predictors of cfPWV values were serum uric acid (B = 0.391, 95%CI 0.321–0.486, *p* = 0.001), fructose load from both fruits and sugar-sweetened beverages (B = 0.310, 95%CI 0.099–0.522, *p* = 0.004), triglycerides (B = 0.228, 95%CI 0.117–0.389, *p* = 0.018), fasting plasma glucose (B = 0.015, 95%CI 0.008–0.022, *p* < 0.001) and estimated Glomerular Filtration Rate (B = −0.043, 95%CI −0.052–−0.035, *p* < 0.001). Conclusion: our data suggest that increased intake of fructose derived from industrial sweetened beverages, though not from fruits, is associated with higher pulse wave velocity.

## 1. Introduction

Diet quality remains a main determinant of morbidity and mortality risk in Western countries [1]. One of the main determinants of a healthy diet is fruit and vegetable intake [2], which is associated with a significantly reduced risk of a number of noncommunicable diseases. Focusing on fruit consumption, a recent meta-analysis of 64 studies, investigating 98 risk–disease pairs showed that for each 100 g/day increase in fruit consumption, the risk ratio was 0.86 (95% Confidence Intervals (CI)0.84 to 0.88) for stroke, and 0.89 (95%CI 0.88 to 0.90) for all-cause mortality [3]. A previous meta-analysis of 95 cohort studies (142 publications) found that relative risk for 200 g/day was 0.90 (95%CI 0.88 to 0.94) [4]. However, fructose intake was associated with a significantly increased risk of cardiovascular diseases [5] and type 2 diabetes [6].

To the best of our knowledge, the association between sugar-sweetened beverage intake and arterial stiffness in the general population has not yet been deeply investigated. Carotid-femoral pulse wave velocity (cfPWV) is a non-invasive measure of the central conduit arteries’ reaction to long-term exposure to the harmful effects of the main cardiovascular risk factors [7]. In the Framingham Study cohort, an increased cfPWV was associated with incident albuminuria (odds ratio per SD 1.28, 95% CI, 1.02–1.61; *p* < 0.05) [8]. A recent meta-analysis of 19 studies concluded that subjects with high cfPWV by 1 standard deviation (SD), 1 m/s, and cutoff points had a high pooled relative risk for cardiovascular events (1 SD: 1.2, 95%CI 1.2 to 1.3; 1 m/s: 1.1, 95%CI 1.1 to 1.2; and cutoff points: 1.8, 95%CI 1.4 to 2.1) and CVD mortality (1 SD: 1.2, 95%CI 1.1 to 1.3; 1 m/s: 1.1, 95%CI 1.0 to 1.1; and cutoff points: 1.8, 95%CI 1.5 to 2.2). cfPWV seemed to be more predictive in subjects at higher baseline cardiovascular risk, compared to those at lower risk [9].

For this reason, the aim of our investigation was to evaluate the association between theintake of fruits and sugar-sweetened beverages, and cfPWV in a large population sample.

## 2. Materials and Methods

The Brisighella Heart Study (BHS) is an epidemiological investigation, carried out on a randomized representative sample of the population of Brisighella, a rural North Italian village. At the baseline, it involved 2939 Caucasian subjects (1491 men and 1448 women), aged between 14–84 and without known cardiovascular diseases at the point of enrolment. The Brisighella Heart Study was carried out in agreement with the declaration of Helsinki and the protocol was approved by the institutional ethical board of the University Hospital of Bologna (Code: BrixFollow-up_1972–2024). All involved subjects signed an informed consent form.

The detailed Brisighella Heart Study protocol was already described in previous reports [10,11]. The standardized operating procedure drawn for the BHS survey visit includes a record of information on personal and family history (with particular interest in lifestyle and pharmacological treatments), a physical examination (including anthropometric data), and records of resting blood pressure (BP) and heart rate, a fasting blood sample and a 12-lead electrocardiogram (ECG).

Waist circumference (WC) was measured at the point of the minimal waist. Body weight was measured with a calibrated precision scale, rounding up readings to 500 g. Height was measured in the standing position, bare feet together and eyes directed straight ahead, and rounded up to the nearest 1cm. Body mass index (BMI) was calculated as weight in kilograms divided by height in squared meters (Kg/m^2^).

The Dietary Quality Index is a validated semiquantitative questionnaire, investigating the usual intake of 18 food items, grouped into three categories, over the past year. The first food group includes complex carbohydrates (i.e., bread, pasta) and fruits/vegetables, the second group encloses animal-derived foods, excepting fish, the third group includes fish, legumes and nuts [12,13]. A further specific question was asked, regarding the consumption of sugar-sweetened beverages.

Systolic (SBP) and diastolic (DBP) BP were measured three times at 1min intervals with a standard calibrated sphygmomanometer, with the subject in the seated position, and after 5 min of quiet rest. The average value of the three measurements was recorded as the individual BP value [14]. EGC results were classified following theMinnesota Code Manual of Electrocardiographic Findings in population studies, which provides a classification of electrocardiographic morphology [15].

The biochemical analyses were carried out on 12h fasting venous blood from the basilic vein. Plasma was obtained by the addition of Na_2_EDTA (1 mg/mL) and centrifuged at 3000× *g* for 15′ at ambient temperature. After centrifugation, the samples were frozen and stored at −80 °C for less than 3 months. The following laboratory parameters were measured with standardized enzymatic-colorimetric methods on semi-automatic analyzer (Cobas C311, Hoffmann-La Roche Ltd., Basel, CH, Switzerland) by the Lipid Clinic Laboratory of the University of Bologna [15]: fasting plasma glucose (FPG), total (TC) and high-density lipoprotein cholesterol (HDL-C), triglycerides (TG), apolipoprotein AI (apoAI), apolipoprotein B-100 (apoB), lipoprotein(a), liver transaminases (alanine aminotransferase–ALT-, aspartate aminotransferase–AST-), glutamyl-transferase (gGT), creatinine, and SUA. All parameters were double-checked. The LDL-C level was calculated with the Friedewald’s formula from TC, HDL-C, and TG concentrations (LDL-C = TC−HDL-C−TG/5). The glomerular filtration rate (eGFR) was estimated with the Chronic Kidney Disease Epidemiology Collaboration equation as a reliable parameter for assessing renal function [CKD-EPI: 141 × Min (creatinine/k, 1)^α^ × Max (creatinine/k, 1)^−1.209^ × 0.9993^Age^ × 1.018 (if female), where *k* is 0.7 for females and 0.9 for males, α is −0.329 for females and −0.411 for males, min indicates the minimum of creatinine/*k* or 1, and max indicates the maximum of creatinine/*k* or 1] [16].

CfPWV was noninvasively measured by the Vicorder^®^ instrument (Skidmore Medical Ltd., Bristol, UK). Vicorder^®^ is a commercially available, validated, operator-independent device, which determines peripheral oscillometric BP using standard cuffs placed around the upper arms and upper legs. All measurements were obtained with the subject in the supine resting position. Brachial pressure waveforms were recorded with the same cuff using a volume displacement technique. Central BP parameters (e.g., augmentation index) were derived from brachial BP waveforms, self-calibrated to brachial SBP and DBP according to a brachial-to-aortic transfer function, described in detail by Hickson et al. [17], and Pucci et al. [18]. The Vicorder^®^ instrument was used in other epidemiological studies, as well [19,20].

**Figure 1:**
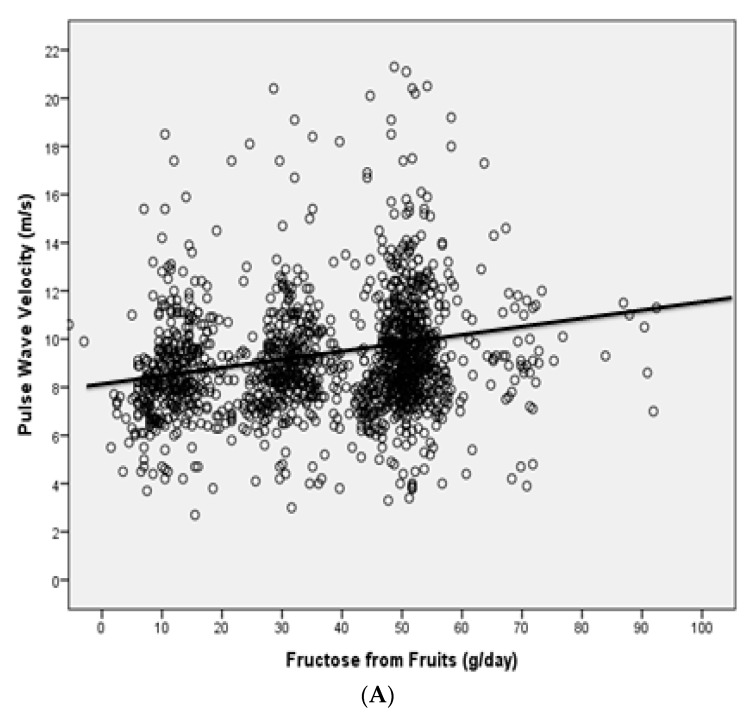

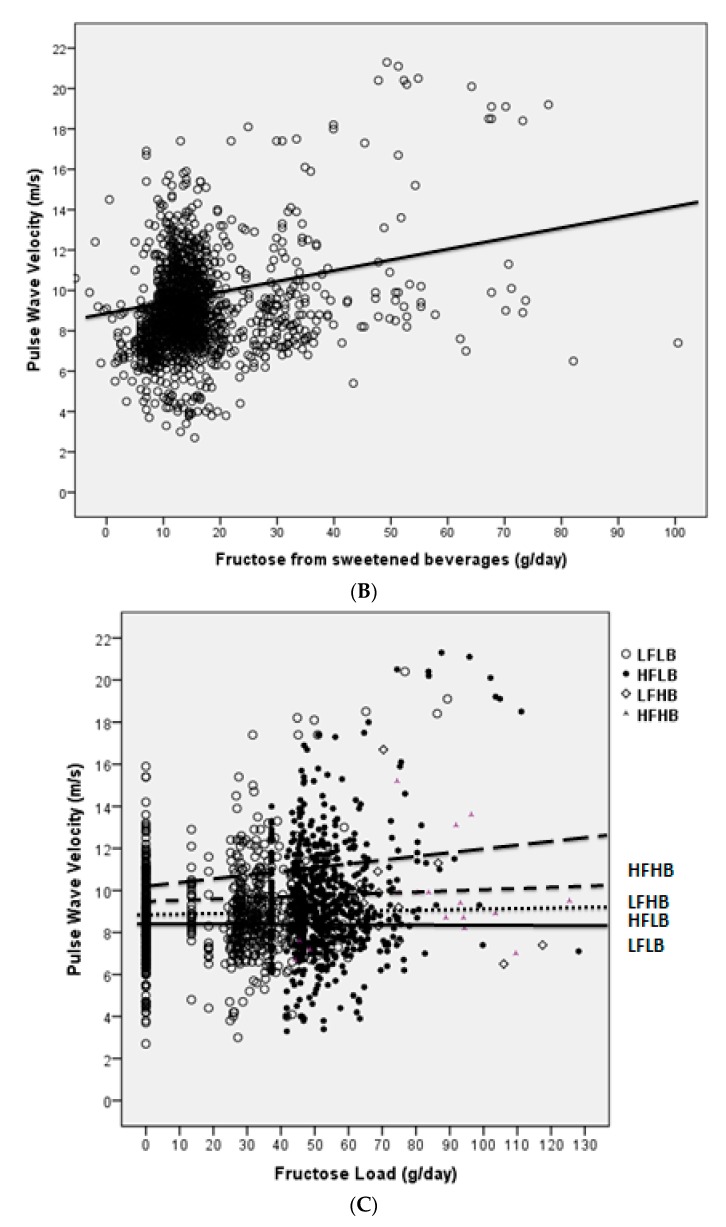
Regression graph plotting (**A**) cfPWV vs. fructose derived from fruits in the whole population sample, (**B**) cfPWV vs. fructose derived from sweetened beverages in the whole population sample and, (**C**) cfPWV vs fructose load in subjects consuming low fructose quantity, high fructose quantity from fruits, high fructose quantity by sugar-sweetened beverages or high fructose quantity from both sources.

For this study, we selected four subgroups of age and gender-matched subgroups from the last Brisighella Heart Study population survey [21], after the exclusion of those in secondary prevention for cardiovascular diseases (coronary artery disease, cerebrovascular disease, intermittent claudication), affected by gout and moderate-to-severe chronic kidney disease (defined as eGFR < 60 mL/min), and/or actively treated with direct vasodilating drugs (calcium-antagonists, alpha-blockers, nitrates) (Figure 1). The remaining subjects were classified in four groups: (1) low fruit and low sugar-sweetened beverage intake (LFLB; n. 437), (2) high fruit and low sugar-sweetened beverage intake (HFLB; n. 419), (3) low fruit and high sugar-sweetened beverage intake (LFHB; n. 133), (4) high fruit and high sugar-sweetened beverage intake (HFHB; n. 116). Low fruit intake was defined as ≤2 portions per day of fresh or juice fruits, low sugar-sweetened beverage intake as ≤1 drink per day.

A full descriptive analysis was performed for the considered variables. A Kolmogorov–Smirnov normality test was carried out for all the continuous variables. All the continuous variables were compared to the considered subgroups of subjects (LFLB, HFLB, LFHB and HFHB) by Analysis of Variance (ANOVA), followed by the Tukey post-hoc test. Non-normally distributed parameters were then log-transformed before continuing with analyses. A univariate analysis was carried out to test factors related to cfPWV. Finally, factors associated with cfPWV were evaluated by a stepwise multiple linear regression analysis, adjusted by age, gender and mean arterial pressure. All tests were carried out with the support of the Statistical Package for Social Sciences (SPSS 23.0) (IBM Corporation, Armonk, NY, USA). A p-value less than 0.05 was considered significant for every test.

## 3. Results

The clinical and laboratory characteristics of the selected subjects are described in Table 1 and Table 2. Gender distribution among subgroups was homogeneous (Table 1). Smokers (~21%), ex-smokers (~16%) and never smokers (~62%) were equally distributed among the study subgroups (Table 2). Similarly, subjects with no/mild (~22%), moderate (~61%) and intense (~17%) physical activity are equally distributed among the study subgroups (Table 1).

Age, DQI score, TC, ApoB, ApoAI, AST, ALT, Creatinine levels and eGFR were similar among the considered subgroups. Compared to the LFLB group, the HFLB group had a significantly higher TG (*p* < 0.05). Subjects with a greater intake of sugar-sweetened beverages (LFHB, HFHB) had significantly higher levels of TG and SUA and lower levels of HDL-C (*p* < 0.05) than ones with lower beverage intake (LFLB, HFLB), independently from fruit intake. Subjects consuming more fruits and sugar-sweetened beverages also had a higher BMI, FPG and gGT (*p* < 0.05) (Table 2).

cfPWV was significantly more elevated in subjects reporting LFLB and HFLB compared with subjects reporting LFHB and HFHB (pooling data: 8.6 ± 2.3 m/s vs. 9.6 ± 2.3 m/s, *p* < 0.001) (Table 3). The individual group comparisons show both HB groups had significantly higher cfPWV than both LB groups, independent of fruit consumption.

In a univariate analysis, the fructose intake from fruits was significantly related to BMI; (r = 0.141, *p* = 0.031), FPG (r = 0.111, *p* = 0.037), and TG (r = 0.289, *p* < 0.001), while fructose intake by sweetened beverages with BMI showed (r = 0.166, *p* = 0.031), FPG (r = 0.122, *p* = 0.032), TG (r = 0.378, *p* < 0.001), SUA (r = 0.487, *p* < 0.001), and gGT (r = 0.144, *p* = 0.033)

Overall, in another univariate analysis, cfPWV was shown to be related to age (r = 0.432, *p* < 0.001), MAP (r = 0.394, *p* < 0.001), BMI (r = 0.138, *p* = 0.034), FPG (r = 0.101, *p* = 0.039), TG (r = 0.388, *p* < 0.001), SUA (r = 0.544, *p* < 0.001), and fructose load from both fruits and sugar-sweetened beverages (r = 0.388, *p* = 0.008), gGT (r = 0.139, *p* = 0.042), and eGFR (r = −0.139, *p* = 0.022).

**Figure 2:**
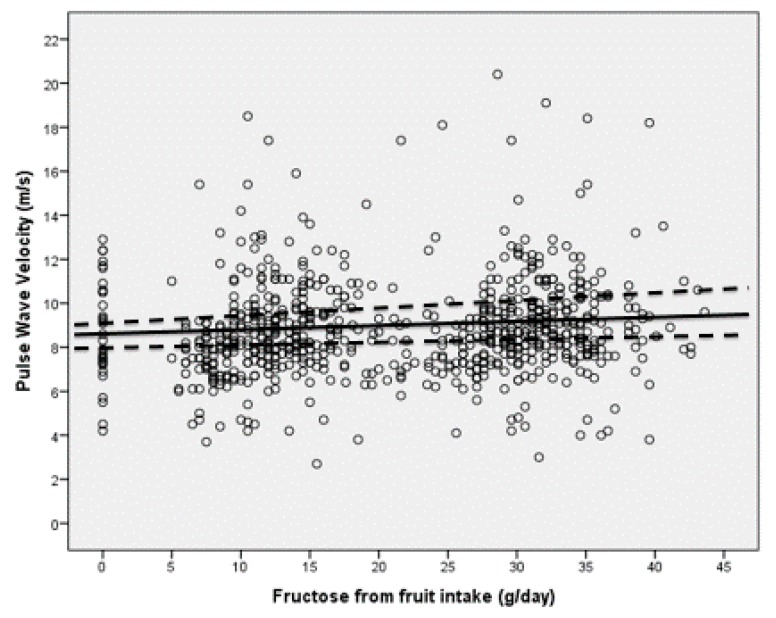
Linear regression between cfPWV and fructose load in subjects consuming high fructose quantity from fruits but low fructose quantity from sugar-sweetened beverages (HFLB).

A regression graph plotting cfPWV vs. fructose load in subjects consuming low fructose quantity, high fructose quantity from fruits, high fructose quantity from sugar-sweetened beverages or high fructose quantity from both sources is reported as Figure 1. A further regression graph showing cfPWV and fructose load of the HFLB group only (Figure 2), has also been plotted: no statistically significant relationship was found between the studied parameters in this group of subjects (beta = 0.173, B = 0.021, 95%CI −0.12 to 0.30, *p* = 0.09).

A stepwise multiple linear regression analysis, adjusted by age, gender and mean arterial pressure, carried out on the whole cohort, shows that cfPWV was significantly predicted by SUA, fructose load from both fruits and sugar-sweetened beverages, TG, FPG and eGFR (protective factor) (Table 4).

## 4. Discussion

In our study, cfPWV was significantly more elevated in subjects consuming a greater fructose load, particularly when it was derived from industrially sweetened beverages. Moreover, the main predictors of cfPWV values were SUA, fructose load from both fruits and sugar-sweetened beverages, TG, FPG and eGFR (protective factor).

Prospective cohort studies reported an association between fructose-containing beverages and weight gain, cardiovascular disease outcomes and type 2 diabetes. Even if increased intake of fructose-sweetened beverages is usually associated with poor lifestyle habits, such as an increased consumption of calories, less exercise, more smoking and a poor dietary pattern [22], in our study, a higher intake of fructose-sweetened beverages was not associated with poor lifestyle patterns.

The higher SBP level in subjects with a higher fructose load from sugar-sweetened beverages could be explained by a direct renal effect of fructose that would induce a salt-dependent BP increase [23]. Our results suggest it is probable that the increased cfPWV observed in subjects consuming fructose from sugar-sweetened beverages was related to the impact of fructose on SUA levels. In previous reports we showed that, in the Brisighella Heart Study cohort, SUA levels were associated with an increased risk in hypertension [24] and arterial stiffness [25]. One of the mediators of the impact of high dietary fructose intake on cardiovascular disease is its relationship with serum uric acid (SUA) levels [26]. A recent meta-analysis of six cross-sectional studies showed that fructose-sweetened beverage intake was associated with 35% greater odds of hyperuricemia (OR: 1.35; 95%CI 1.19 to 1.52) [27]. This could be partly mediated by the insulin-resistance associated with fructose intake, and also by the metabolization of the adenosine monophosphate (AMP), by the AMP deaminase, to inosine menophosphatase and SUA [28]. However, fructose load induces hepatic de novo lipogenesis [29,30], consequently increasing TG levels and potentially affecting arterial stiffness, as demonstrated by our data. However, fruit components beyond fructose (Vitamin C, epicatechin, flavonols, potassium and fibers) could counteract the negative effects of both fructose and SUA [31]. Similarly, in prospective cohort studies, fructose-sweetened beverage consumption was associated with an increased risk of gout (relative risk = 2.08, 95%CI 1.40 to 3.08), while fruit intake was not [32]. In the Bogalusa Heart Study, serum fructose was associated with increased cfPWV [33]. These finding are in line with our observation that fructose-sweetened beverage intake is directly related tocfPWV. Of course, in observations in models adjusted for uric acid and TG, intake of fructose-sweetened beverages is still significantly associated with PWV, suggesting that additional mechanisms of action exist, through which beverage fructose increases PWV. They will require further investigation.

Our investigation has several limitations. Firstly, the sample size of the considered subjects is relatively small. However, the selected subsample was age- and sex-matched with the global Brisighella Heart Study cohort, thus remaining representative of the original cohort. Secondly, the transversal design of the study does not allow us to test for a cause–effect relationship. Theselection of a subgroup of subjects with specific characteristics reduces the possibility of inferring obtained results to unselected populations. However, we think that excluding all subjects in secondary prevention for cardiovascular diseases, those affected by gout and moderate-to-severe chronic kidney disease, and/or those actively treated with direct vasodilating drugs from the study reduced the possibility of cfPWV being modified by different factors than the ones we were investigating. However, other factors related to sweet intake could have influenced arterial stiffness, such as insulin-resistance, which was not estimated in our study. The administration of the Dietary Quality Index, given only at the time of the visit, is another limitation of the analysis, since itofferedno information about the subjects’ eating habits over time, although the population of Brisighella has a dietary pattern which is homogeneous and constant over time, seen in a previous educational intervention carried out on the Brisighella population [34]. However, the questionnaire was validated based on a 12 month recall and, in a large cohort, was able to predict the development of an unfavorable cardiometabolic profile [35]. Finally, the dietary questionnaire used in our study could not detect fructose sources other than the ones derived from fruits and sugar-sweetened beverages. However, it is probable that all the four considered subgroups consume those fructose sources in a similar amount, because of the high homogeneity of dietary habits of Brisighella citizens [36,37].

## 5. Conclusions

In conclusion, our data suggest that increased intake of fructose derived from industrial sweetened beverages, though not from fruits, seems to be harmful, being independently associated with higher pulse wave velocity. However, further research is required to clarify if a permanent decrease in the intake of fructose-sweetened beverages could be associated with an improvement in arterial function and/or slowing of arterial aging processes.

## Figures and Tables

**Table 1:** Categorical variable distribution in the studied population subgroups.

	LFLB (N. 437)	HFLB (N. 419)	LFHB (N. 133)	HFHB (N. 116)
Gender				
- Males	48%	55%	57%	57%
- Females	52%	45%	43%	43%
Smoking habit				
- Current	21%	19%	22%	21%
- Former	18%	17%	16%	15%
- Never	61%	64%	62%	64%
Physical activity				
- No/Mild	20%	21%	24%	22%
- Moderate	63%	61%	61%	60%
- Intense	17%	18%	15%	18%

LFLB = low fruit and low sugar-sweetened beverage intake, HFLB = high fruit and low sugar-sweetened beverage intake, LFHB = low fruit and high sugar-sweetened beverage intake, HFHB = high fruit and high sugar-sweetened beverage intake.

**Table 2:** Age, BMI, dietary and laboratory characteristics of the studied population subgroups (values reported as mean ± standard deviation).

	LFLB (N. 437)	HFLB (N. 419)	LFHB (N. 133)	HFHB (N. 116)
Age (years)	59.6 ± 15.6	61.9 ± 14.7	59.1 ± 16.8	58.7 ± 16.8
Dietary Quality Index score	39.8 ± 4.6	41.4 ± 4.4	38.9 ± 4.9	39.0 ± 5.0
Fructose intake from fruits (g/day)	6.6 ± 3.5	38.7 ± 9.7 *	8.5 ± 3.8	38.4 ± 11.5 *^#^
Fructose intake from sugar sweetened beverages (g/day)	7.1 ± 3.7	2.5 ± 2.6 *	35.3 ± 12.2 *	34.2 ± 12.9 *°
Body Mass Index (kg/m^2^)	26.4 ± 4.5	26.7 ± 4.7	26.8 ± 4.0	27.1 ± 4.6 *
Total Cholesterol (mg/dL)	216.8 ± 16.1	224.8 ± 15.3	226.3 ± 20.1	229.1 ± 20.7
Triglycerides (mg/dL)	140.44 ± 45.0	157.07 ± 46.0 *	167.24 ± 42.0 *°	183.19 ± 48.6 *°^#^
HDL- Cholesterol (mg/dL)	52.4 ± 5.9	51.2 ± 4.5	46.2 ± 3.9 *°	44.6 ± 4.8 *°^#^
LDL- Cholesterol (mg/dL)	137.8 ± 16.2	143.3 ± 17.7	147.6 ± 23.3 *	146.8 ± 20.5 *
Apolipoprotein AI (mg/dL)	154.6 ± 18.9	153.1 ± 17.2	144.5 ± 16.1	144.6 ± 29.2
Apolipoprotein B (mg/dL)	90.82 ± 7.4	93.7 ± 7.1	94.5 ± 6.5	93.3 ± 6.9
Fasting Plasma Glucose (mg/dL)	91.5 ± 9.7	92.7 ± 9.7	94.7 ± 10.0	95.9 ± 14.2 *
AST (U/L)	23.5 ± 4.9	23.5 ± 4.9	21.1 ± 4.1	23.2 ± 4.9
ALT (U/L)	25.8 ± 4.6	23.4 ± 4.4	22.8 ± 4.0	28.0 ± 5.0
gamma-GT (U/L)	27.7 ± 5.0	26.3 ± 8.1	27.5 ± 9.4	40.7 ± 9.0 *°^#^
Serum Uric Acid (mg/dL)	5.3 ± 1.3	5.1 ± 1.2	5.7 ± 1.4 *°	5.9 ± 1.5 *°
Creatinine (mg/dL)	1.04 ± 0.17	1.03 ± 0.22	1.06 ± 0.19	1.03 ± 0.16
Estimated GFR (mL/min)	73.4 ± 5.2	74.0 ± 5.4	73.7 ± 6.1	74.5 ± 5.5

* *p* < 0.05 vs. LFLB; ° *p* < 0.05 vs. HFLB; ^#^ *p* <0.05 vs. LFHB. LFLB = low fruit and low sugar-sweetened beverage intake, HFLB = high fruit and low sugar-sweetened beverage intake, LFHB = low fruit and high sugar-sweetened beverage intake, HFHB = high fruit and high sugar-sweetened beverage intake.

**Table 3:** Haemodynamic characteristics of the studied population subgroups (values reported as mean ± standard deviation).

	LFLB (N. 437)	HFLB (N. 419)	LFHB (N. 133)	HFHB (N. 116)
SBP (mmHg)	139.3 ± 11.5	138.1 ± 12.2	142.5 ± 11.3 *°	142.0 ± 15.5 *°
DBP (mmHg)	734 ± 5.8	73.6 ± 5.7	74.5 ± 5.7	72.3 ± 6.3
Heart Rate (bpm)	64.0 ± 12.9	63.8 ± 10.4	66.4 ± 12.2	63.0 ± 10.1
cfPWV (m/s)	8.8 ± 2.1	8.4 ± 2.5	9.5 ± 2.4 *°	9.8 ± 2.2 *°

* *p* < 0.05 vs. LFLB; ° *p* < 0.05 vs. HFLB. LFLB = low fruit and low sugar-sweetened beverage intake, HFLB = high fruit and low sugar-sweetened beverage intake, LFHB = low fruit and high sugar-sweetened beverage intake, HFHB = high fruit and high sugar-sweetened beverage intake, SBP = Systolic Blood Pressure, DBP = Diastolic Blood Pressure, cfPWV = carotid-femoral Pulse Wave Velocity, m/s = meter per second.

**Table 4:** Parameters associated with pulse wave velocity in age, gender and mean arterial pressure adjusted models (stepwise multiple regression analysis; the beta coefficient is the degree of change in the outcome variable for every 1-unit of change in the predictor variable).

		95%CI		
Parameter	Beta Coefficient	Lower Limit	Upper Limit	Sig,
Model 1 (Including fruit-derived fructose)				
Fasting Plasma Glucose(mg/dL)	0.018	0.006	0.025	0.003
Triglycerides(mg/dL)	0.132	0.079	0.272	0.021
Fruit-derived fructose	0.184	0.091	0.302	0.009
Model 2 (Including sugar-sweetened beverages)				
Estimated Glomerular Filtration Rate(mL/min)	−0.055	−0.074	−0.031	<0.001
Fasting Plasma Glucose(mg/dL)	0.019	0.007	0.028	0.001
Serum Uric Acid(mg/dL)	0.389	0.229	0.492	<0.001
Triglycerides(mg/dL)	0.233	0.115	0.394	0.012
Sugar-sweetened beverages	0.295	0.096	0.535	0.003
Model 3 (Including fructose load from both fruits and sugar-sweetened beverages)				
Estimated Glomerular Filtration Rate(mL/min)	−0.043	−0.052	−0.035	<0.001
Fasting Plasma Glucose(mg/dL)	0.015	0.008	0.022	<0.001
Serum Uric Acid(mg/dL)	0.391	0.321	0.486	0.001
Triglycerides(mg/dL)	0.228	0.117	0.389	0.018
Fructose Load	0.310	0.099	0.522	0.004
